# Factors Contributing to School Effectiveness: A Systematic Literature Review

**DOI:** 10.3390/ejihpe13100148

**Published:** 2023-09-30

**Authors:** Špela Javornik, Eva Klemenčič Mirazchiyski

**Affiliations:** Educational Research Institute, Gerbičeva 62, 1000 Ljubljana, Slovenia; eva.klemencic@pei.si

**Keywords:** school effectiveness (research), SER, school performance/outcomes, systematic literature review

## Abstract

This paper aims to provide a systematic review of the literature on school effectiveness, with a focus on identifying the main factors that contribute to successful educational outcomes. The research question that this paper aimed to address is “what are the main factors of school effectiveness?”. We were interested in several descriptors such as school, effectiveness/efficiency theories, effectiveness/efficiency research and factors. Studies (published within the 2016–2022 period) were retrieved through two databases: JSTOR and ERIC. This paper defines several categories identified by school effectiveness research. Within these categories, various factors that affect the students’ outcomes and the defined effectiveness in school are listed. As the results show, the issue of school effectiveness is multifaceted, as the effectiveness of schools is a complex concept that can be measured through various indicators such as academic achievement, student engagement and teacher satisfaction. The review of school effectiveness revealed that several factors contribute to effective schools, such as strong leadership, effective teaching practices, a positive school culture and parental involvement. Additionally, school resources, such as funding and facilities, can impact school effectiveness, particularly in under-resourced communities.

## 1. Introduction

The answer to the question “what makes school effective?” is the Holy Grail of educational research [1]. School effectiveness has been a research topic for several decades, with scholars and policymakers seeking to identify the key factors that contribute to successful educational outcomes. The concept of school effectiveness refers to the extent to which a school is able to achieve its goals and objectives in terms of student learning, development and well-being [2]. This article is not focused on the historical view of school effectiveness research (SER) or on phases in its development but rather on identifying factors that contribute to school effectiveness. School effectiveness research concerns educational research and explores differences within and between schools and malleable factors that improve school performance [3] and/or achievements and/or outcomes. Educational (school) effectiveness can be defined as the degree to which an educational system and its components and stakeholders achieve specific desired goals and effects [4]. Taking into consideration the different terminology used in researching school effectiveness and that we were not focusing on those possible differences when describing our results, let us first focus on possible differences to which effective schooling can contribute—as the specific desired goals and effects of schooling can be numerous and especially because different aspects of those goals can be inter-linked.

Schools have important “effects” on children and their development; so, “schools do make a difference”, as stated by Reynolds and Creemers in [5] (p. 10). SER studies seek to include factors such as “gender, socio-economic status, mobility and fluency in the majority language used at school” in assessing the impact of schools [5] (p. 11). In the past, educational assessment mainly relied on basic metrics like the number of students advancing to higher education, the grade repetition rates and special education enrollment. However, it became clear that these metrics were influenced by external factors beyond school and teacher characteristics and were thus abandoned. Instead, more comprehensive measures focusing on academic achievement in subjects like math and language were introduced. Progress in assessing effectiveness continued with the inclusion of control measures, such as students’ prior knowledge and family socio-economic status. Presently, standardized objective tests are the primary tool for measuring educational effectiveness in specific curricula [4].

In recent years, there has been a growing interest in understanding the factors that contribute to school effectiveness, particularly in light of concerns about the quality of education and the need to improve educational outcomes. Research suggests that school effectiveness is a multifaceted concept that is influenced by a range of factors, including school leadership, teacher quality, curriculum and instruction, school culture and climate, parental involvement and student characteristics [2,6,7]. However, the relative importance of these factors may vary depending on the context in which they are examined. Therefore, it is important to conduct a comprehensive review of the literature to identify the key factors of school effectiveness across different contexts.

This paper aims to provide a systematic review of the literature on school effectiveness, with a focus on identifying the main influencing factors. The review drew upon a range of empirical studies, meta-analyses and reviews to provide a comprehensive overview of the current state of knowledge on this topic. In this research, the literature review was conducted according to the PRISMA (Preferred Reporting Items for Systematic Reviews and Meta-Analyses) guidelines [8]. Systematic reviews of the literature have an important role and can identify different problems that can be addressed in future studies, “they can generate or evaluate theories about how or why phenomena occur’’ and can address questions that cannot be tackled by individual studies through several studies [8] (p. 1). We were interested in several descriptors such as school, effectiveness/efficiency theories, effectiveness/efficiency research, and factors. Studies were reviewed using two databases: JSTOR and ERIC. This paper defines several categories that are important in school effectiveness research and, within these categories, lists various factors that affect students’ outcomes and the defined school effectiveness. The research question that this paper aimed to address is “what are the main factors of school effectiveness?”. This paper can be helpful as it provides an overview of school effectiveness research, and the research question is of significant importance, as answering it can help inform educational policy and practice by identifying the key areas that schools should focus on in order to improve student outcomes. Several studies attempted to answer this question, but there is still much debate and discussion surrounding the factors that contribute to school effectiveness.

## 2. Background of School Effectiveness Research

The concept of school effectiveness emerged in the 1960s and 1970s in response to growing concerns about the quality of education and the need to improve educational outcomes for students [4,7,9]. Early definitions of school effectiveness focused on the achievement of educational outcomes, such as academic performance and the ability of schools to meet the needs of students from diverse backgrounds [4,10].

Coleman et al. [11] argued that students’ socioeconomic status is a crucial factor affecting their academic achievement in schools and has a greater impact than school characteristics. This is consistent with the conclusion reached by Jencks [12], who found that schools do not have a statistically significant impact on student achievement. These findings paved the way for school effectiveness research, which emerged in the early 1970s as a radical movement aimed at exploring the factors within schools that contribute to better students’ educational performance, regardless of their social background [13]. In the field of education, effective-schools research emerged as a response to previous studies such as Coleman’s and Jencks’, which indicated that schools had little impact on students’ achievement. As titles such as “Schools can make a difference” and “School matters” suggest, the goal of effective-schools research was to challenge this notion and explore factors that contribute to successful schools. What sets effective-schools research apart is its focus on investigating the internal workings of schools, including their organization, structure and content, in order to identify characteristics associated with effectiveness [14,15]. According to Muijs [14], school effectiveness research sought to move beyond the prevailing pessimism about the impact of schools and education on students’ educational performance. The movement aimed to focus on studying the factors within schools that could lead to better students’ academic performance, irrespective of their social background [14] (p. 141). Scheerens et al. [16] (p. 43) summed up the five influencing factors identified in early research on school effectiveness: “strong educational leadership, emphasis on the acquiring of basic skills, an orderly and secure environment, high expectations of pupil attainment, frequent assessment of pupil progress”.

According to various scholars [17,18,19,20,21,22], defining educational quality is a challenging task due to the diverse settings, stakeholders and goals involved in education. Generally, educational quality can be defined as achieving the desired standards and goals. Creemers and Scheerens [23] further added that quality refers to the characteristics and factors of the school that contribute to differences in outcomes between students in different grades, schools and educational systems. However, these definitions fail to provide a clear explanation of the specific characteristics that result in quality education and schools [4] (p. 2). School effectiveness is a subset of educational effectiveness or educational quality. According to Scheerens [21], educational effectiveness refers to the extent to which an educational program or institution achieves its intended outcomes, while school effectiveness is concerned with the extent to which a school achieves its goals and objectives. Burusic et al. [4] also noted that school effectiveness research is a branch of educational effectiveness research that specifically focuses on the functioning of schools and their impact on student outcomes.

Theories of school effectiveness have evolved over time, with a greater emphasis on the role of leadership and school culture in shaping educational outcomes. One of the most influential models of school effectiveness is the “Effective Schools Model” developed by Edmonds [24]. This model identified five key characteristics of effective schools: high expectations, strong instructional leadership, a safe and orderly environment, a focus on basic skills, and frequent monitoring of student progress.

Subsequent research confirmed the importance of these factors in promoting school effectiveness [2,25]. For example, a study by Leithwood et al. [2] found that effective school leadership was associated with improved student outcomes, including academic achievement and graduation rates. Similarly, research by Ismail et al. [26] highlighted the importance of a positive school culture, including supportive relationships among staff and students, in promoting school effectiveness.

Reynolds et al. [27] (p. 3) proposed that there are three primary areas of focus in School Effectiveness Research (SER):

- School Effects Research: investigating the scientific characteristics of school effects, which has evolved from input–output studies to current studies that use multilevel models.
- Effective Schools Research: researching the procedures and mechanisms of effective schooling, which has developed from case studies of exceptional schools to contemporary studies that integrate qualitative and quantitative methods to study classrooms and schools concurrently.
- School Improvement Research: examining the methods through which schools can be transformed, utilizing increasingly advanced models that surpass the simple implementation of school effectiveness knowledge to employ sophisticated “multiple-lever” models.

Sammons and Bakkum [5] (p. 10) argued the importance of different factors that are associated with student attainment: “individual characteristics (age, birth weight, gender), family socio-economic characteristics (particularly family structure, parental background: qualification levels, health, socio-economic status, in or out of work, and income level), community and societal characteristics (neighborhood context, cultural expectations, social structural divisions especially in relation to social class)”.

More recent theories of school effectiveness also emphasized the need to address systemic inequities and promote social justice in education [28,29]. These theories recognize the role of societal factors, such as poverty and discrimination, in shaping educational outcomes and the need for schools to adopt a more inclusive and equitable approach to education. For example, Ainscow [28] developed a model of “inclusive school leadership”, which emphasizes the importance of creating a culture of inclusion and diversity in schools.

Overall, theories of school effectiveness have evolved over time, reflecting changing perspectives on the role of schools in promoting educational outcomes. Key factors identified in the literature include effective school leadership, a positive school culture and a focus on meeting the needs of diverse students. However, more recent theories also recognize the need to address systemic inequities and promote social justice in education.

According to Heyneman and Loxley [30], multiple linear regression was used to re-analyze IEA data on student achievement in industrialized countries. The researchers found that student background variables such as parental education, father’s occupation, number of books at home, use of a dictionary at home, the sex of the student and the age of the student explained approximately 20% of the total variance in science achievement, which accounted for roughly 50% of the explainable variance. Furthermore, the OECD [30] reported that PISA 2000 also revealed that various student background factors, such as parental occupational status, cultural possessions at home, parental involvement, home educational resources, participation in cultural activities and family wealth, explained the significant variance in the academic achievement of 15-year-old students.

## 3. Materials and Methods

For this article, a systematic literature review was carried out. The literature review was conducted using the PRISMA protocol [8]. “To ensure a systematic review is valuable to users, authors should prepare a transparent, complete, and accurate account of why the review was done, what they did (such as how studies were identified and selected), and what they found (such as characteristics of contributing studies and results of meta-analyses)” [8] (p. 1). We were interested in several descriptors such as school, effectiveness/efficiency theories, effectiveness/efficiency research and factors. For searching, the following formula was used: (school AND effectiveness) OR (school AND efficiency)) AND (theories OR research OR factors).

Two databases were used: JSTOR and ERIC. The search and review of the studies were carried out from August to October 2022. The period was limited between 2016 and 2022, except for the database ERIC, as we did not have that option. In the ERIC database, we examined research within the last five years, from 2018 to 2022, which was one of the options in the database. In JSTOR, the period was limited between 2016 and 2022. This decision was made because this literature review will be used in further research for a doctoral dissertation of the main authors of this article, where a secondary analysis will be performed considering the ICCS 2016/2022 (International Civic and Citizenship Education Study); therefore, we focused on the literature in that period.

The literature review included all studies in English, qualitative and quantitative. There were no specific restrictions on the studies involved, so book sections and articles published in professional and academic journals were considered.

Before we determined the final search formula, we tried several search terms and combinations. The search using the term “school effectiveness” was too broad, and, for example, that using the terms “school effectiveness theories” or “school effectiveness factors” was too narrow. We were also interested in the term efficiency, besides the term effectiveness; so, the following final search term was chosen: (school AND effectiveness) OR (school AND efficiency)) AND (theories OR research OR factors).

In the first phase of searching, we included descriptors and searched the literature using the final formula mentioned above.

Both databases have different options for searching studies, which is the reason why searching was individually adapted to our interests. With the already mentioned search formula, we obtained 130,371 results. The resources to which we did not have full access were excluded, and the final number of relevant items decreased to 13,446.

**Figure 1:**
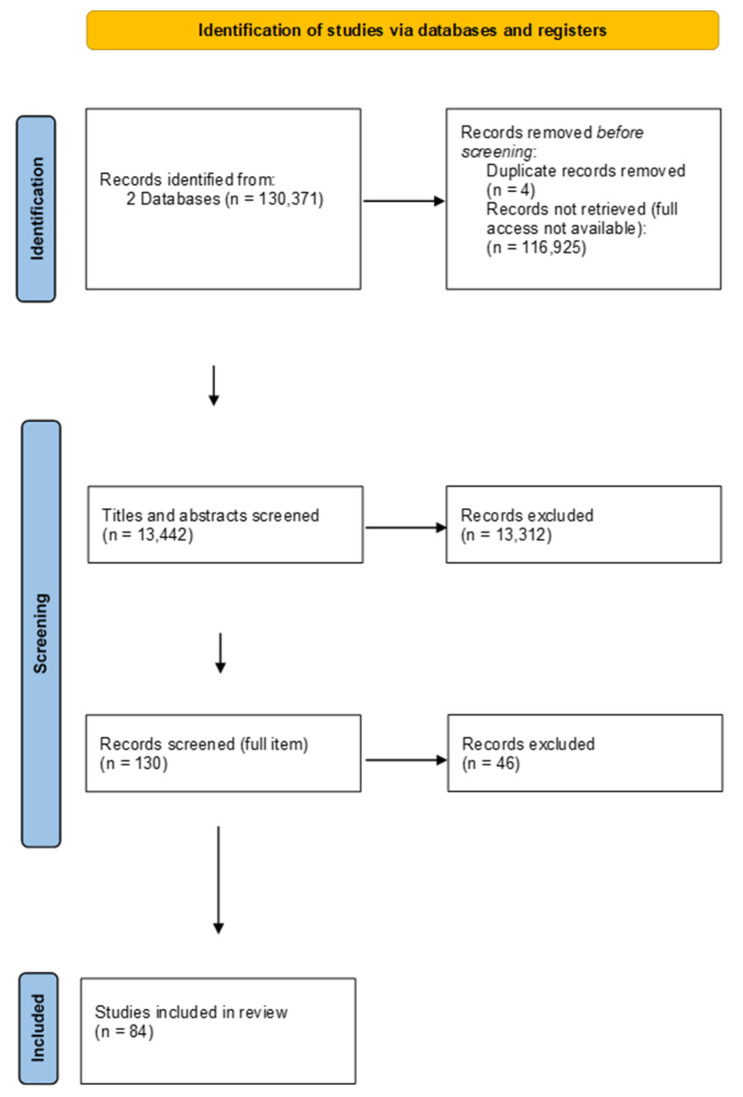
PRISMA diagram for the search protocol and the inclusion and exclusion of the reviewed articles.

In the second phase of the literature review, we reviewed all the titles of the searched items and collected 130 possible relevant studies for our research area. We excluded 4 duplicates. After we read all selected articles, we excluded the irrelevant ones, and the final number of included studies in the systematical literature review was 84. The description of those articles is in the Results section. For a more visual picture of the search process for the literature review, please refer to the PRISMA diagram in Figure 1.

## 4. Results

With the literature review in the area of school effectiveness, we identified key themes and provide theoretical guidance for the achievement of effective schools. The aim of this study was to discover and define the key factors that influence the effectiveness of a school and students’ achievements/outcomes. A few categories were identified within school effectiveness research: teacher effectiveness, effectiveness in digital/online education, and school efficiency. In the different items that we reviewed, some key factors that appeared to have a statistically significant impact on school effectiveness and student achievement were identified in several studies. Factors such as school culture, a supportive climate in the classroom, a positive class climate, the use of digital sources, a strong and firm leadership, an effective leadership, flipped classroom (FC), schools’ economic, social and cultural status, the attitude of principals, teachers, and school counselors, the organizational climate, the aspects of cooperation, inclusion in decision making, presence of teachers with many years of experience, collegial support, collegial leadership, teacher collaboration, the level of participation in decisions, the willingness to participate, the habit of treating students with respect and caring about their problems, high teacher ratings on leadership and the supervisor’s support of teachers were all revealed as important contributors to overall school effectiveness and student achievement.

The majority of the reviewed studies mostly discussed school-level factors. Thrupp in [3] argued that the background characteristics of the students are often overlooked. “School performance is usually expressed in terms of average student achievement by the school” [3], (p. 255). Furthermore, research suggests that “student achievement mostly depends on the performance of the student in early education” [31] (p. 12). School climate was detected as one of the most important factors for school effectiveness [32,33,34,35,36,37,38], and studies indicated the significance of school climate for teacher commitment [33,39,40,41].

### 4.1. Positive School Climate and School Culture

A positive school climate is essential for school effectiveness. Khan [33] proposed that it would be worthwhile to develop a positive organizational climate strategy to improve teacher commitment, and promoting a positive school climate is important for the improvement of school effectiveness in general [34]. Authors like Ismail et al. [34] and Ismara et al. [42] claimed that improving school effectiveness requires support from stakeholders like government, policymakers, principals, deputy principals, teachers, parents and other school stakeholders.

Also, school culture predicts school effectiveness and has stronger relations with school effectiveness than teacher empowerment [43]. “A school should have a culture that values the professional development of its teachers, collegiality, collaborative leadership, and teamwork to be effective” [43] (p. 340). Karadağ et al. [44] argued that high-performing schools have strong school culture and spiritual leadership characteristics compared to low-performing schools. The results of their study showed the impact of school culture and spiritual leadership on academic success. Ismail et al. [26] also claimed that school culture has a significant influence on school effectiveness. “If school leaders want to shape a new culture, they should start with an assessment of the climate. If the culture is ineffective, there are probably climate issues that were missed before they became rooted in the culture” [45] (p. 58). The school should have a culture that “values the professional development of its teachers, collegiality, collaborative leadership, and teamwork to be effective” [43], (p. 340).

### 4.2. Teacher Effectiveness

Teacher effectiveness is also known as one of the most important factors for predicting school and student effectiveness [31,46,47,48,49,50]. Factors not significant in explaining differences in teacher effectiveness estimates are student gender and students’ language identity, as Aslantas claimed [31].

Effective teachers provide a positive school climate, collaborate with colleagues and analyze student data. Student achievement is positively associated with years of teaching experience [49]. The quality of the interactions between teachers and students is also very important. LoCasale-Crouch et al. [51] argued that teacher–student interactions are important to students’ school outcomes (they affect their engagement, academic performance and motivation). Independent of the overall interaction quality, students with less consistency in their interactions with teachers had more conflicts with them.

School effectiveness is positively correlated with the teachers’ level of participation in decisions and their willingness to participate. Teachers reported that they did not feel enough included in the decisions of the administration and were aware that the administration had an important role. It is therefore very important to increase the level of participation of teachers in decisions [52]. Yıldırım [53] claimed that organizational cynicism (OC) indirectly affects perceived school effectiveness (PSE) through involvement in the decision-making (IDM) process and may reduce perceived school effectiveness by reducing teachers’ participation in school decision making. OC had a statistically significant negative effect on PSE, as well as on IDM. IDM showed a statistically significant positive effect on PSE [53]. Gülbahar [54] (p. 15) reported that “the perceived supervisor support among teachers is positive on school effectiveness perception, engagement to work and job satisfaction and negative on organizational cynic attitude”.

Javorcíková et al. [55] analyzed the motivation level of teachers in primary schools. The supervisors’ approach as well as the atmosphere in the workplace, teamwork, fair system and salary are important for teachers’ positive motivation. Khan [33], on the other hand, tested the impact of organizational climate on teachers’ commitment. School climate is directly connected with school effectiveness, and Khan researched how it is associated with teacher commitment. He performed a regression analysis and argued that the school climate has a significant influence on teacher commitment. Also, collegial leadership and institutional vulnerability appeared as predictors of teacher commitment. Teacher professionalism and academic achievement failed to be predictors of teacher commitment. The study proposed that it would be worthwhile to develop a positive organizational climate strategy to improve teacher commitment [33].

### 4.3. Strong Leadership

Many authors agree that strong instructional, school, academic, collaborative and collegial leadership has a significant influence on the effectiveness of schools [32,33,34,36,39,41,42,43,44,48,56,57,58,59,60,61,62,63,64,65,66,67,68].

Reynolds and Teddlie in [32] (p. 2) “summarized that effective schools were characterized by nine process factors: effective leadership, effective teaching, a pervasive focus on learning, a positive school culture, high expectations for all, student responsibilities and rights, progress monitoring, developing school staff skills, and involving parents”.

Professional development is a dimension of school culture. Gülşen and Çelik [43] tested the correlation with school effectiveness, and professional development was the most predictive variable. The other significant predictors were collegial support, collegial leadership, unity of purpose, self-efficacy, decision making and teacher collaboration. The following variables were not statistically significant in explaining school effectiveness: the learning partnership dimension of school culture and the status, impact, autonomy, and professional growth dimensions of school participant empowerment.

### 4.4. Technological Resources and Digital Literacy

This systematic literature review included many articles that discussed technological resources and digital literacy as important factors that can be effective in providing positive effects on education. It is necessary that teachers receive more support and training on using digital resources in education. Teachers partly use digital sources, and most of them do not consider it a workload. Teachers see the usage of digital sources as motivating student engagement in education and as having a positive effect on student success and education [69,70].

The studies examined in this systematic literature review also focused on online education and distance teaching and learning, since there was the COVID-19 pandemic during the considered research period; so, we identified some issues related to education during the COVID-19 pandemic [71,72,73,74]. The experience of online distance learning (ODL) was new for the students and teachers, and they faced some issues related to the lack of tools and sources and poor Internet connectivity to access virtual classes. Despite some difficulties, the teachers and students reported that ODL has many positive aspects that help working teachers and professionals to continue higher education and professional development [71].

Zou et al. [72] found that it is important for teachers who have the opportunity to continue their training to acquire more skills and become more confident, so their online teaching could be more effective. In general, in this study, the majority of students and teachers were satisfied with online teaching during the pandemic and reported that in general it was effective. Basar et al. [73] argued that tools and sources were not a problem for the students, as they had computers and an internet connection, and their ability and comfort to use computers were high. The main problem for the students was the lack of motivation for online learning. The majority of the participants in the study agreed that face-to-face teaching is very important. The authors also emphasized “the importance of well-equipped facilities and a stable internet connection for effective learning” and that the “support within school communities, and among parents and school administrators, is vital to ensure the success of online learning”. “While online learning has been proven to support the health of students during the pandemic, it is not as effective as conventional learning” [73] (pp. 76, 119, 128).

However, online education is nowadays more included in school systems; so, we must increase the effectiveness in that area of learning. The teachers who were educated beforehand and used the technology before the pandemic were more self-assured and had fewer problems with the transition [72,74,75,76].

### 4.5. Flipped Classroom

In our systematic literature review, we found that a few authors researched the learning method of flipped classroom and tested its association with school effectiveness [77,78,79,80,81].

Flipped classroom is a strategy of active learning that puts the student at the center of teaching and that gained popularity in the recent decade. Authors like Mok and Gilboy et al. in [79] argued that compared to traditional pedagogical teaching, students positively accept the strategy of a flipped classroom with more enthusiasm and motivation for learning. On the other hand, Atteberry [79] reported in a preliminary study that, according to four professors, flipped classroom did not improve students learning, though the difference was not significant. The flipped classroom (FC) method is a digital teaching method according to which the courses are taught online through learning applications and are supported by digital media, for example, learning videos and simulations [77].

Weiß and Friege [77] (p. 315) listed several definitions of the flipped classroom concept, from different authors:

- “An inverted (or flipped) classroom is a specific type of blended learning design that uses technology to move lectures outside the classroom and uses learning activities to move practice with concepts inside the classroom” Strayer (2012, p. 171).
- “We define the flipped classroom as an educational technique that consists of two parts: interactive group learning activities inside the classroom and direct computer-based individual instruction outside the classroom” Bishop and Verleger (2013, p. 9).
- “Flipped Learning is a pedagogical approach in which direct instruction moves from the group learning space to the individual learning space, and the resulting group space is transformed into a dynamic, interactive learning environment where the educator guides students as they apply concepts and engage creatively in the subject matter” Association of Flipped Learning Network (2014, p. 1), Bergmann and Sams (2014, p. 14)”.

There are not yet clear conclusions on whether the method of the flipped classroom contributes to school effectiveness. This strategy of learning brings many benefits and, on the other hand, students and teachers face new challenges, and students have to be well organized for self-learning and for learning at home. Although research on that theme has grown in the last few years, there is a lack of publications and of relevant publications that meet the scientific standards [77].

Flipped classroom is an effective method that increases students’ engagement, and most students prefer this method of learning. But, on the other hand, it also has some disadvantages. The strategy did not further improve the scores of top-scoring students. Though the students did not prefer this method to the traditional one, it “helped improve the grades of students who were at the lower end of academic performance” [78] (p. 2). Knežević et al. [81] found a positive relationship between the method and school effectiveness, reporting that the strategy of flipped classroom brings higher results than the approach of conventional teaching and learning.

### 4.6. The Efficiency of Schools

The articles we examined for our systematic literature review reported on the efficiency of schools [71,82,83,84,85,86]. This concept includes the financial status of the school (budget), the number of employed staff at the school (teaching staff) and the school’s physical infrastructure [87]. “Efficient educational institutions are those that can use their inputs optimally to achieve maximum possible outputs. If the output is fixed, efficiency refers to minimizing the use of inputs to achieve the output” [87] (pp. 1, 2).

The study by Thompson et al. [82] showed that total student enrolment is a significantly essential and positively affecting factor for the efficiency rating of school districts. The percentage of nonwhite students and of economically disadvantaged students has a significant negative influence on the district efficiency scores. The most used method for measuring technical efficiency involves comparing inputs and outputs in many educational units. This method is called “Data Envelopment Analysis” (DEA) and was developed by Charnes et al. in [85] (p. 2). Halkiotis et al. [85] measured the degree of technical efficiency of high schools, and they found that it is necessary to improve the working conditions for teachers and reduce stress. The study results showed that a significant number of teachers did not complete their compulsory weekly teaching schedule. Furthermore, it is essential to develop a healthy competition between the students by increasing the average number of students per class.

### 4.7. Sociodemographic Characteristics

The sociodemographic characteristics have been identified as important factors that contribute to school effectiveness. Ramberg and Modin [56] found that schools with a high proportion of students with immigrant backgrounds tended to have lower levels of academic achievement, possibly due to language barriers and cultural differences. Şirin and Şahin [88] also noted that students from low-income families may face more challenges in their academic performance and school engagement, which can negatively impact the overall effectiveness of schools.

However, Hirschl and Smith [89] argued that the relationship between socioeconomic status and school effectiveness may not be straightforward, as schools in high-poverty areas may have higher levels of student motivation and community involvement, which can offset the negative effects of poverty. Murwaningsih and Fauziah [90] further highlighted the importance of considering gender and ethnicity to understand school effectiveness, as these factors can influence student achievement and experiences in different ways.

A study by Rumberger et al. [91] analyzed data from the National Longitudinal Survey of Youth and found that socioeconomic status was a strong predictor of high school graduation rates. The study also found that students from low-income families were more likely to attend schools with fewer resources, which might contribute to lower academic achievement.

Overall, these studies suggest that the sociodemographic characteristics play a significant role in school effectiveness, but their influence may vary depending on specific contexts and populations.

## 5. Discussion

Academic performance, sometimes known as school readiness, academic achievement and school performance are often used as synonyms, and several authors agree that it is the result of learning, prompted by the teaching activity of the teacher and produced by the student [92]. However, at the same time, there seems to be a lack of consensus among researchers regarding the similarities and differences among the constructs of academic performance, achievement, and learning outcomes [93]. For those who view them as the same concept, they can be used interchangeably. But for others—who mostly come from different disciplines and thus have various knowledge regarding the perception and the ways each of these constructs were used in relation to certain variables—they can have different meanings [93], although to a low extent. The academic performance of a student can be regarded as the observable and measurable behavior of a student in a particular situation and can be evaluated through the scores obtained in teacher-made tests, first term examinations, mid-semester tests, etc. [93], which can be measured at any point. Achievement is a measurable behavior in a standardized series of tests, as indicated by Simpson and Weiner in [93] (p. 6), or is measured by a standardized achievement test developed for school subjects, as indicated by Bruce and Neville in [93] (p. 6). This means that academic achievement is measured in relation to what is attained at the end of a course, since it is the accomplishment of a medium- or long-term objective of education (cannot be attained within a short period or in one instance). It is important that the test should be standardized to meet the national norm [93] (pp. 6–9). Academic achievement is a representation of performance outcomes that indicate the level to which the student has attained specific goals that were the focus of activities in instructional environments [94]. School systems mostly define cognitive goals that either apply across multiple subject areas (e.g., critical thinking) or include the acquisition of knowledge and understanding in a specific intellectual domain (e.g., numeracy, literacy, science, history). Therefore, academic achievement should be considered as a multifaceted construct that comprises different domains of learning [94]. The definition of academic achievement depends on the indicators used to measure it. Among the many criteria that indicate academic achievement, there are very general indicators (e.g., procedural and declarative knowledge acquired in an educational system), more curricular-based criteria (e.g., grades or performance on an educational achievement test), and cumulative indicators of academic achievement (e.g., educational degrees and certificates) [94]. Academic achievements are usually expressed through school grades, as reported by Martinez-Otero in [92]. Learning outcomes may be used when looking for performance or achievement as an attitude of the students towards a particular subject [93] (p. 14). Aremu and Sokan [95] indicated that learning outcomes (academic achievement and academic performance) are determined by family, schools, society and motivation factors.

In summary, it appears that academic outcomes (performances and/or achievements) or educational goals and effects are influenced by several school and out-of-school (e.g., family) factors, as well as by student (individual) factors, with inter-relation factors also being of importance. And all of this contributes to school efficiency.

As shown in the previous section (Results), there are many factors contributing to school effectiveness. Some of them can be seen as related to each individual student (e.g., sociodemographic characteristics); however, also those “individual factors” are often directly or indirectly associated with within-school factors which are attributed to teachers and school efficiency (e.g., effective teaching, effective school or classroom leadership, etc.). And this is not a surprise, as Creemers and Kyriakides [96] already proposed that a new, dynamic model of effectiveness must (a) be multilevel in nature, (b) assume that the relation of some effectiveness factors with achievement may be curvilinear, (c) illustrate the dimensions upon which the measurement of each effectiveness factor should be based and (d) define relations among the effectiveness factors. Their testing of this dynamic model at the school (and, further, system) level placed most attention on describing detailed factors associated with teacher behavior in the classroom [96].

The subject of school effectiveness is a complex and multifaceted topic that has been the subject of extensive research and debate. There are many different factors that can contribute to the effectiveness of a school, including the quality of teaching, the study curriculum, the leadership and management of the school, the socio-economic background of the students, the level of parental involvement, the school culture and the school climate. The quality of the school seems to be strongly linked to a safe and stimulating learning environment. There is not a clear division in the definition of a safe and of a stimulating learning environment; furthermore, the concepts of a safe learning environment and of a stimulating learning environment are complementary and partly overlap with the concept of school culture and climate, as indicated by Dumont et al. in [97]. Standards defining the school culture and climate or a safe learning environment, highlight the following aspects: “inclusion, safety, relationships, information and communication, educational strategies” [97], (p. 9).

One important finding that emerged from the literature is that the quality of teaching is a very important factor in school effectiveness. Research has consistently shown that effective teaching practices can significantly improve student outcomes, including academic achievement, engagement and motivation [98,99]. This underscores the importance of ensuring that teachers have the necessary skills, knowledge and support to deliver high-quality instruction.

Another key finding is that school leadership and management can have a significant impact on school effectiveness. Effective leadership can create a positive school culture that promotes learning and growth, fosters collaboration among staff and students and ensures that resources are allocated effectively [2,100]. The socio-economic background of the students is also an important factor to consider when evaluating school effectiveness. Research has shown that students from disadvantaged backgrounds are more likely to experience academic difficulties and that schools that serve these populations face unique challenges [101]. In order to be effective, the schools must be able to provide these students with the support and resources they need to succeed. Important factors in the growth of students’ academic success (university students) are their sociodemographic characteristics, variables such as gender, the university where they studied, their fathers’ education and the way they chose their department [33].

## 6. Limitations of Our Systematic Review

There are several limitations regarding the review processes used in this literature review and the evidence reported. One limitation is the possibility of a publication bias. This review only included studies that were published in peer-reviewed journals and reported only in two databases, and we only included items to which we had full access. Another limitation is the potential for methodological differences across the studies. The studies included in this review used a variety of research methods and comprised case studies, surveys, and quantitative analyses, which could have resulted in variations in the findings. Furthermore, the studies may have used different definitions of school effectiveness or different measures of school inputs and outputs, which could make it difficult to compare findings across studies. Many of the studies included in the review relied on self-reported data, which may have introduced bias and inaccuracies in the findings. This review was limited by its focus on English-language studies published from 2016. This may have excluded relevant studies published in other languages or earlier than 2016. Additionally, the rapid pace of change in education policies and practices means that this review may not reflect the most up-to-date research in the field.

## 7. Conclusions and Future Directions

In conclusion, the effectiveness of schools is a complex and multifaceted concept that can be measured through various indicators such as academic achievement, student engagement and teacher satisfaction. This review of school effectiveness revealed that several factors contribute to effective schools, such as strong leadership, effective teaching practices, a positive school culture and parental involvement. Additionally, school resources, such as funding and facilities, can impact school effectiveness, particularly in under-resourced communities.

Leadership is a crucial factor in promoting student success, as noted by multiple researchers [2,48,67,102,103]. Leaders who create a positive school culture and prioritize high-quality teaching practices are more likely to create a learning environment where students can thrive. Furthermore, parental involvement has been linked to improved student achievement, as noted by Akbar et. al. [104] and Fan and Chen [105], and can be facilitated through strategies like family engagement programs and clear communication between families and schools.

Teacher quality is another critical factor in student learning and achievement [106,107,108]. Teachers who are knowledgeable, experienced and effective in using instructional strategies can significantly impact student outcomes. Effective curriculum and instruction, aligned with standards and assessments and delivered using evidence-based instructional strategies, are critical in promoting student learning and achievement [109,110].

Moreover, the inclusion of all students regardless of their backgrounds and abilities can promote a sense of belonging and engagement, which can positively impact their academic performance and social-emotional development, as noted by Ahn and Davis [111]. Additionally, a positive school climate is essential for promoting student learning and achievement. A safe, respectful and supportive school environment can positively impact student outcomes [33,38,112].

Finally, adequate resources, including funding, facilities and technology, are essential for promoting student learning and achievement, as noted by Bhutoria and Aljabri [87]. It is important to note that school effectiveness is a complex and multifaceted concept and that different factors may be more or less important depending on the context. Answering the research question, the literature suggests that effective schools are characterized by strong leadership, high-quality teachers, effective curriculum and instruction, parent and community involvement, a positive school climate and adequate resources. These factors work together to create a supportive learning environment that promotes student learning and achievement. However, by studying and understanding the key factors that contribute to school effectiveness, educators and policymakers can work to create environments that promote student success and support all students in reaching their full potential. By implementing evidence-based practices and strategies that prioritize strong leadership, effective teaching practices, parent and community involvement, a positive school culture and adequate resources, schools can provide high-quality education that meets the needs of all students. Overall, the literature suggests that school effectiveness is a multidimensional concept that requires a comprehensive and holistic approach to be achieved. By understanding the various factors that contribute to school effectiveness and implementing evidence-based practices, schools can provide high-quality education that meets the needs of all students.

While this literature review provides valuable insights into the factors that contribute to school effectiveness, its findings should be interpreted with caution, given the limitations of the review processes used. Future research in this area should consider addressing these limitations and building on the findings of this review to provide a more comprehensive understanding of school effectiveness. Despite these limitations, this literature review provides a valuable summary of the current research on school effectiveness and efficiency, highlighting the key factors that contribute to these outcomes. Future research could build on these findings by addressing some of the limitations of this review, such as conducting more comparative studies across different contexts and using consistent measures of effectiveness and efficiency.

## Data Availability

No new data were created. Results are based on existing articles on the topic.
